# Southern Surgical and Gynæcological Association

**Published:** 1892-12

**Authors:** 


### *SOCIETY REPORTS.*

---

### SOUTHERN SURGICAL AND GYNECOLOGICAL ASSOCIATION.

---

*Fifth Annual Meeting, held in Louisville, Kentucky, November* 15, 16 *and* 17, 1892.

---

FIRST DAY—MORNING SESSION.

The Association met in the council chamber of the city hall, and was called to order at 9 : 30 A. M. by the President, DR. J. MCFADDEN GASTON, of Atlanta, Ga.

An address of welcome was delivered by Dr. L. S. McMurtry, of Louisville, Chairman of the Committee of Arrangements, the response to which was made by the President.

The first paper read was by Dr. Bedford Brown, of Alexandria, Va., entitled,

PERSONAL RECOLLECTIONS OF THE LATE DR. BENJ. W. DUDLEY, OF LEXINGTON, KY., AND HIS SURGICAL WORK.

The speaker paid an eloquent tribute to Dr. Dudley, and characterized him as the greatest lithotomist that this country has ever produced and the most successful in the history of the world. The speaker's close relationship to Dr. Dudley as private pupil and assistant for two years enabled him to present a clear and faithful sketch of his character and surgical work.

EXPERIENCES IN PELVIC SURGERY.

This was the title of a paper read by Dr. A. V. L. Brokaw, of St. Louis, Mo. Of all the surgical problems difficult to solve, it may be truthfully said, that those met with in the pelvis are the most trying. The speaker knew of no surgical work which will compare with the experiences found in the pelvis ; a diversity of conditions, complications and unexpected happenings are ever presenting. In a series of many operations but few will be alike in every particular. As his experience became larger he was free to confess his inability to correctly diagnose the character of abdominal and pelvic troubles. He had diagnosed pus tubes and found extra-uterine pregnancy ; diagnosed extra-uterine pregnancy and found pus ; diagnosed ovarian lesions and found the trouble located in the tubes and *vice versa*. When well defined pelvic lesions exist nothing short of radical measures succeed. The one condition above all others where exploratory incision should be adopted was in cases of suspected extra-uterine pregnancy. It was correct and good surgery to open the abdomen and not wait for all the classical signs to appear. The symptoms of extra-uterine pregnancy were so frequently obscure and unreliable that he was firmly convinced a radical position should be taken. A case was cited in point.

DR. WILLIAM WARREN POTTER, of Buffalo, desired to indorse that portion of the paper pertaining to an early exploratory incision in cases of suspected extra-uterine pregnancy. As regards the use of the sound he had brought an indictment against it some six or eight years ago, consequently he would not expatiate upon the subject at this time.

DR. JOSEPH TABER JOHNSON, of Washington, said that as soon as the surgeon diagnosed something in the abdominal cavity that ought not to be there, anatomically or physiologically, and was histologically wrong, it should be removed. An exploratory operation was justifiable in cases of suspected extra-uterine pregnancy, and the surgeon should base his further procedures upon what he finds after making the exploration.

DR. W. E. B. DAVIS, of Birmingham, Alabama, thought the pendulum relative to surgical interference had swung a little too far. He believed that a great many of the so-called "tinkerers," who succeeded in relieving their patients, did not accomplish it so much by the local treatment they used as by having patients under their care, keeping the bowels open, giving constitutional treatment, seeing them regularly, etc. While by so doing they may not be cured in all cases, they were greatly benefited. Regarding the diagnosis, surgeons who are opening the abdomen constantly would rarely give a positive diagnosis in the case. Dr. Davis cited the case of a woman who had an acute attack of peritonitis, and the history was the same as from pelvic abscess.

DR. BROKAW, in closing the discussion, said that in every case of suspected extra-uterine pregnancy it was good surgery to make an exploratory incision and operate before rupture took place.

DR. CORNELIUS KOLLOCK, of Cheraw, South Carolina, read a paper on

### CRANIOTOMY UPON THE LIVING FŒTUS NOT JUSTIFIABLE.

He said this operation implied the death of the fœtus and a frightful mutilation of its body, often accompanied by serious lacerations of the vagina and adjacent tissues of the mother. Recent advances in obstetrics, gynecology and abdominal surgery contribute largely to a demonstration of the fact that a timely resort to Cæsarean section in pelvic obstruction is the great factor to success. In Germany, out of 149 cases of contracted pelvis, 109 mothers and 136 children were saved. If craniotomy had been done in those cases 149 children would have been destroyed and probably 50 women—perhaps more, making a sacrifice of at least 199 lives. In many of these cases exhaustion had supervened and septic influence had already been excited. This, added to a tardy disposition to union by first intention, caused by contusion of the parts involved in the uterine incision, lessened materially the woman's chances for recovery. Zweiffel was successful in twenty-nine cases out of thirty ; Schauta did Cæsarean section fifteen times without a single death. Recently in eighteen operations done in Louisiana fourteen were successful. Of eight in Ohio six were successful. Dr. Price has done Cæsarean section a number of times successfully. Dr. Kollock is firmly convinced that eighty-five or ninety-five per cent. of the cases of obstruction of the pelvis forbidding the delivery of the fœtus in the natural way, might be saved by a timely resort to the Cæsarean section.

DR. W. D. HAGGARD, of Nashville, emphasized the position taken by Dr. Kollock. He believes that when the profession fully realizes the immense difference in the number of lives saved by Cæsarean section over craniotomy there will be no doubt as to its preference to the latter operation.

DR. HUNTER McGUIRE, of Richmond, favored Cæsarean section. Some time ago he saw the report of a case by Dr. Thomas of New York, where, in doing Cæsarean section, he proposed to take the uterus out of the cavity, and then open it. He thought this added very much to the danger of the operation, necessitating a larger opening, exposing the cavity of the abdomen a long time to the atmosphere, etc. He does not favor this procedure.

DR. L.S. McMURTRY, of Louisville, said that a few years ago it would have been impossible for one to have presented the views that Dr. Kollock had without meeting with violent opposition. Cæsarean section was then regarded as an extremely heroic operation, and until recent years the mortality therefrom was very great ; but since it has been carried to the present degree of perfection by Sænger and others it has strengthened the opinions of abdominal surgeons, who now consider it preferable to craniotomy. Within the last two months symphysiotomy had been brought before the profession and practiced as an alternative in certain cases for Cæsarean section. What the future of the former operation is to be, we are not prepared to say.

DR. ARCH DIXON, of Henderson, Ky., advised Cæsarean section in a case in which he was called in consultation, but the family physician insisted upon his doing craniotomy, which was done, and while every precaution was taken with regard to rendering aseptic the field of operation, the woman developed pelvic peritonitis, and died within four days. He believed a Porro operation would have saved the life of the woman, and perhaps that of the child.

DR. W. D. HAGGARD, of Nashville, read a paper entitled

A CASE OF EXTENSIVE HEMATOCELE RESULTING FROM TUBAL PREGNANCY RUPTURING INTO THE BROAD LIGAMENT.

Although the fœtus was not found, that it was a case of tubal pregnancy with rupture into the broad ligament is clearly established by the clinical history and post mortem appearances, summarized as follows: (1) Patient confessed having had intra-pelvic trouble previously (presumably gonorrhœa), for which she was treated locally. (2) At the time of the accident, caused by jumping from a wagon, her menses were past due. As to how long her statements were misleading. (3) There was a fitful yet persistent bloody flow from the uterus during her entire illness. (4) Paroxysmal, colicky pains in lower abdominal and pelvic regions of frequent occurrence. (5) Existence of a tumor above the pubes, which she probably mistook for a gravid uterus. (6) Persistent refusal to submit to a digital examination, probably fearing the detection of her pregnant state.

*Post Mortem Appearances.*—(a) Enlarged and softened condition of the uterus with a patulous os, showing escape of a sero-sanguinal, stringy fluid. (b) Enlargement of the left tube with a well-defined cavity from which the fruit sac escaped. (c) Existence of a deciduous membrane, as revealed by the microscope. (d) Discoloration of rectum, produced by blood dissecting around it, producing constriction and partial death.

Dr. H. C. Hogan, of Union Springs, Ala., reported a case of

FIBROID TUMOR OF THE UTERUS—PREGNANCY: RUPTURE ABOUT THE FOURTH MONTH—OPERATION, SPECIMEN.

The woman, colored, was twenty-eight years of age, and from the symptoms and history of the case, he was satisfied there was a rupture, and the probabilities were that it was about the fourth month of gestation. He was also of the opinion that the rupture did not immediately destroy the fœtus, that it continued to grow in its abnormal position. The speaker felt sure that if he had operated on the case immediately after rupture, the patient's life would have been saved. In all cases of rupture he would advise Porro's operation to be done immediately; that in all cases, where the tumor is large or multiple, intramural or subperitoneal, with a sacciform dilatation of the posterior segment of the uterus, and as above the pubic bone, or inaccessible, the same operation should be done. In all cases where the tumor is in front of the child, or blocking the passage, it should be done, provided the pregnancy has advanced to the full time, or there should be a hemorrhage, or rupture of the membranes, indicating that an abortion or miscarriage is imminent.

---

FIRST DAY—AFTERNOON SESSION.

Dr. Geo. A. Baxter, of Chattanooga, Tenn., read a paper entitled

A NEW OPERATION FOR THE RADICAL CURE OF INGUINAL HERNIA.

Dr. Baxter presented a radically different operation in principle from any yet given. If consists in a prolongation of the incision, after the ordinary management of the sac and after ligation, through the internal ring into a more or less extensive laparotomy as the exigencies of the case demand; lifting the neck of the sac into the abdominal opening above the ring and its fixation there by a deep suturing, cutting off the sac close above the peritoneum and its closure by buried suture, and a final closure of the abdominal opening by this and a more superficial set of sutures which pass across above the closed sac and peritoneum and underneath the deep fasciæ which are intended to approximate the homologous tissues of the abdominal wall. The ring is closed with crucial sutures dipping over cord and traversing the tissues, and the seminal canal closed with deep sutures alone.

Points of originality claimed: A line of incision suitable for any inguinal hernia, and by the fixation of the sac above the peritoneum a deflection of all abdominal expulsive force from the ring and canal, and the thickened lining of the internal ring, and the method of closure of abdominal incision. Advantages claimed: Quick cure with avoidance of necessity of truss, deflection of expulsive force from internal opening and canal to abdominal parietes. Advantage in being able to approach constriction either from without or within. Avoidance of necessity for traction on sac or contents. Ample room for treatment in diseased conditions of sac or contents, including gut operations if necessary.

Dr. Henry O. Marcy, of Boston, followed with a paper on

THE CURE OF INGUINAL HERNIA IN THE MALE, in which he said until recently the cure of inguinal hernia in the male had been considered at the best accidental, and when apparently effected, generally doubtful, and the hernia liable to return. The great majority of surgeons looked upon an attempt to cure as ill-advised, and believed operative measures should not be undertaken except in cases of strangulation. Dr. Marcy thought there was abundant reason for such conclusion, when judged from the earlier history of surgical procedures as attempted for cure. The essential surgical considerations for the cure of hernia were as follows: First—Strict aseptic conditions. These pertain alike to all modern surgical procedures. Second—A free dissection. This is necessary in order to lay bare the internal ring, to permit of the enucleation of the peritoneal sac, and the separation and elevation of the cord out of the wound. The external epigastric artery often courses in the line of the incision. It is not seldom that the size of this vessel is such that the operator fears he has wounded the larger vessel. Third—The disposition of the sac. The separation of the sac to its very base before removal is to be recommended as the rule. There are times, however, when it is not easy to free the peritoneal pouch, owing to the adhesion to the surrounding tissues, and in large old irreducible hernia a more or less intimate fusion of the contents to the inner wall of the sac. It is generally better to open the sac before ligating or sawing through its neck, since by so doing the condition at the internal ring is assured, and by such knowledge the operator is often profited, even if the sac is completely empty, not seldom the omentum is adherent at the internal ring and even a constriction loop of intestine may escape observation when it is attempted to return the sac unopened.

Dr. Marcy closed his paper by saying that between three and four millions of the people living in the United States were subject to hernia ; and, if the demonstration is complete that the risk of life is less than one per cent. from the operative procedures instituted for cure, and that scarcely more than ten per cent. are subject to relapses, and these almost invariably in a state improved by the operation, the plea is a very strong one, to consider favorably the advisability of operation in a very large majority of all the sufferers from hernia.

DR. W. H. WATHEN, of Louisville, read a paper entitled

THE TREATMENT OF UMBILICAL AND VENTRAL HERNIA.

He said the importance of studying carefully the best methods of treating hernia is now especially emphasized because of the increased frequency of the disease following laparotomy, and especially because the modern methods of surgery make the operation far less dangerous than it formerly was. The operation for radical cure of hernia in the practice of the best surgeons, except in extreme cases, is practically devoid of danger, and the result may be made permanent. Modern antiseptic and aseptic precautions have practically excluded the danger which formerly arose from infective peritonitis. The author said there are many cases of ventral hernia that could have been prevented had the proper treatment been carried out in the closure of the abdominal wound. In order that there may be no hernia following laparotomy it is necessary to get perfect union by adhesion of all the layers of tissue forming the abdominal wall—the peritoneum, muscles, the deep and superficial fascia, and the skin. But especially must we get union of the layers of fascia, for unless this be done the other layers will gradually separate and hernia will follow. This cannot be done unless we succeed in bringing the cut edges of the fascia in even and perfect apposition long enough for strong union to occur.

---

SECOND DAY—MORNING SESSION.

DR. W. O. ROBERTS, of Louisville, read a paper on

THE TREATMENT OF UNUNITED FRACTURES BY RESECTION.

He said the treatment of ununited fractures by resection was more than a hundred years old, White of Manchester having done the first operation in 1760. In consequence of the great mortality attending the operation it was abandoned until revived by Sir Benjamin Brodie. In 1805 Horeau, after having divided the fragments obliquely fastened them together by tying a metallic wire around them. Rogers, of New York, in 1838 passed the wire through holes drilled in the wall of the fragment and then twisted it. Since then other surgeons have used sutures of various materials in the same way. Some of them leaving the sutures in permanently, while others removed them after union of the fragments had occurred. Some instead of drilling the bone passed the sutures simply through the periosteum. Screws, nails, ivory pegs and clamps have been used for the same purpose. In the long bones when coaptation of the fragments can be secured, Dr. Roberts feels satisfied that resection and a fixed dressing will be followed by just as good results as when sutures or other contrivances for fastening the ends of the fragments together are used.

"Symptoms of Fracture, their Importance and Significance." Dr. W. C. Dugan, of Louisville, read a paper on this subject. (Abstract of same not received.)

DR. BEDFORD BROWN, of Alexandria, Va., related the case of a boy who sustained a compound comminuted fracture of the skull in 1860, yet he was perfectly conscious and had no symptoms of compression of the brain. The spiculæ of bone were removed, and recovery followed.

DR. J. H. LETCHER, of Henderson, Ky., advised against the too hasty resort to the use of the trephine and chisel in injuries of the skull.

DR. C. KOLLOCK, of Cheraw, S.C., had trephined in two cases with successful results.

DR. J. H. MCINTIRE, of St. Louis, reported a case of traumatic insanity in a railroad employee in which he trephined with success. The fracture was an extensive one and occurred in the upper Rolandic region. He reported several other interesting cases.

DR. WILLIAM WARREN POTTER, of Buffalo, called attention to fracture of the internal table of the skull without fracture of the external; hence the great liability to error in diagnosis.

DR. HOWARD A. KELLY, of Baltimore, related the case of a man who fell and was brought to the Presbyterian Hospital in a comatose state. Careful examination revealed the fact that the man had diabetic cataract with fracture at the base of the skull.

DR. WILLIAM T. BRIGGS, of Nashville, had trephined in fifty cases of epilepsy. Four-fifths of the cases operated on were relieved temporarily but not permanently.

DR. T. W. REAMY, of Cincinnati, mentioned the case of a man who had fallen from the second story of a court house, sustaining a fracture of the skull, but had never had epilepsy or any bad symptom following the injury. He thought this case would be some comfort to country practitioners who did not enlarge the scalp wound in all cases.

The papers were further discussed by Drs. Vance, Lydston, Nicolson, Greenly and Baxter, all of them favoring radical measures in the treatment of injuries of the skull.

DR. L. S. McMURTY, of Louisville, read a paper entitled "Ovarian Cystoma with Twisted Pedicle and Peritonitis; Ovariotomy in Second Week of Typhoid Fever—Recovery." (No abstract received of this paper.)

DR. H. HORACE GRANT, of Louisville, contributed a paper on

INTESTINAL ANASTOMOSIS BY A NEW DEVICE.

For more than a year the speaker has been endeavoring to perfect some instrument to simplify suture, but it has been so difficult to get just what he wanted that time has not been allowed since the completion of the instrument to test it fully. It is to be used only after resections. The two blades of the clamp are oval scissors, one-fourth of an inch in transverse and two and a half inches in longitudinal diameter. The arms of the clamp are made long enough to allow of introduction full five inches. After the gut is exposed, a strand of iodoform gauze is passed through the mesentery and constricts the intestines fully six inches from each point of intended resection. The mesentery is tied off over the portion to be resected with fine silk, in two inch loops, cut close and dropped in the usual way. When the resected portion is removed the gut ends may be washed out if desired. While the two ends of the divided intestine are held parallel, one blade is entered in each, allowing at least one and one-half inches of gut beyond the proposed anastomotic opening to permit of invagination of the ends. The clamp is tightened and the two surfaces thus firmly held are rapidly stitched together by a continuous overhand Lembert suture of fine silk. Two rows of parallel sutures as suggested by Abbe may be used if desired, though it has seemed that one is enough according to the author's experiments. The work can be done far more rapidly and accurately than without the clamp. When the suturing is finished the clamp is tightened if need be, and a long bladed dressing forceps passed in the bowel and the oval plug removed or pushed in. The scissors action of the blades, together with the ten or fifteen minutes pressure prevents any hemorrhage. The clamp is now withdrawn and the ends invaginated in the usual way.

Dr. A. V. L. Brokaw, of St. Louis, thought the instrument exhibited by Dr. Grant a good one, and said that anything which materially assists the surgeon in making intestinal anastomotic operations rapidly was of great value, that time was a most important element. The use of rings, plates and mats in the past were bad. He believes that we can suture far more rapidly with Dr. Grant's instrument than any other device he has thus far seen.

Dr. W. E. B. Davis, of Birmingham, Ala., believed a large number of operators had abolished mechanical devices in doing intestinal anastomosis. His brother, Dr. John S. Davis, had devised a rubber plate and mat, but now prefers not to use the plate. In the case of restriction of the bowel, he thought the device of Dr. Grant was an ingenious one, inasmuch as it would facilitate the work of the surgeon and enable him to do an operation very quickly. He had conducted a series of experiments in an effort to do away with mechanical devices, by which surgeons might use the end to end operation by splitting up the bowel. While the operation was successful in some cases, the strain on the circulation was too great, and he now condemns the operation.

Dr. Frank G. Lydston, of Chicago, directed attention to Dr. J. B. Murphy's anastomosis button, a recent device by which he says cholecysto-enterostomies can be done in from eight to twelve minutes.

[TO BE CONCLUDED.]

